# Supplementary material for: SPINK2 deficiency causes infertility by inducing sperm defects in heterozygotes and azoospermia in homozygotes
Source: EMBO Mol Med. 2017 May 29;9(8):1132–49. doi: 10.15252/emmm.201607461 (PMC5538632; doi:10.15252/emmm.201607461)

Appendix - Table of content

Appendix Table S1. List of homozygous variants present in B1 and B2.

Appendix Table S2. List of *SPINK2* variants identified in azoo- and oligo- spermic patients.

Appendix Table S3. Sequence of the primer used for *SPINK2* sanger sequencing.

Appendix Table S4. Sequence of the primer used in RT-PCR and size of the amplified products

Appendix Table S5. Sequence of the primer used in real time RT-PCR and size of the amplified products

Appendix Table S6. Sequence of the primer used for genotyping and size of the amplified products.

Appendix Figure S1. Expression of *SPINK2* mRNA in different organs in human and mouse.

Appendix Figure S2. Impact on the protein structure of the c.206-3C>G *SPINK2* variant

Appendix Figure S3. Absence of *Spink2* expression in testicular extracts from KO mice.

Appendix Figure S4. Specificity of antibodies targeting SPINK2.

Appendix Figure S5. Absence of Spink2 does not lead to DNA fragmentation in spermatogenic cells.

Appendix Figure S6. Equal protein loading was verified by stain-free gel technology and Western blots against house keeping protein.

Appendix Table S1. List of homozygous variants present in B1 and B2.
Coordinates of all variations are based on the UCSC GRCh37/hg19 assembly. [&] No RefSeq transcript accession number currently available. Nucleotidic references

Gene	Variant coordinates	Transcript	cDNA Variation	Amino acid variation	Prediction	Testis expression
SPINK2	Chr4:57686748G>C	NM_001271718.1<br>NM_001271720.1<br>NM_001271722.1<br>NM_021114.3	Splice acceptor site	-	Damaging	High and predominant
GUF1	Chr4:44683156G>T	NM_021927.2	c.637G>T	p.Ser148Ile	Damaging	Low
ZCCHC5	ChrX:77913158A>G	NM_152694.2	c.1056A>G	p.Phe254Leu	Damaging	Low
ARR3	ChrX:69498482C>G	NM_004312.2<br>ENST00000374495[&]	c. 947C>G<br>c.994C>G	p.Ser299Cys	Damaging	Low
FTHL17	ChrX:31089629TC>AA	NM_031894.2	c.542C>A	p.Glu148Leu	Damaging	low

Appendix Table S2. List of *SPINK2* variants identified in azoo- and oligo- spermic patients (RefSeq : NM_021114**).**

Coordinates of all variations are based on the UCSC GRCh37/hg19 assembly. [&] No RefSeq transcript accession number currently available. The 5'UTR was only sequenced in 363 patients and the whole coding sequence was sequenced in all 611 patients . When the variant was not identified in ExAC the number of ExAC controls indicated corresponds to the number of subject analyzed in ExAC for the nearest described variant. We note that the beginning of *SPINK2* exon1 is particularly poorly covered.

Position	Localisation of the variation	cDNA reference	Consequences on the protein	Nb of homozygous patients (%)	Nb of ExAC homozygous controls (%)	Nb of heterozygous patients (%)	Nb of ExAC heterozygous controls (%)
4:57687851 (rs115163565)	5' UTR	c.1-23 A>T	-	2/363 (0,55)	307/4980 (6,2)	32/363 (8,8)	1816/4980 (36,5)
4:57687849 (rs114591157)	5' UTR	c.1-21 G>C	-	3/363 (0,83)	318/5065 (6,3)	30/363 (8,3)	1852/5065 (36,0)
4:57687828	Exon 1	c.1 A>T	p.M1L	0/611	0/6054	1/611	0/6054

Appendix Table S3. Sequence of the primer used for *SPINK2* sanger sequencing.

Primer name	Primer sequence	Size of amplicon
SPINK2-hSeq-Ex1-F	GAGTGGCGCAGGTAACAGAC	245 bp
SPINK2-hSeq-Ex1-R	CTGGGGAACCGCCAGTAAC	
SPINK2-hSeq-Ex2-F	TGGCTAATGCCTCAAATTCC	339 bp
SPINK2-hSeq-Ex2-R	ACGCAGTCCTCAATGGTTTC	
SPINK2-hSeq-Ex3-F	ACACGGTGAAACCCTGTCTC	552 bp
SPINK2-hSeq-Ex3-R	CAGAGGTTGCAGTGAACCAA	
SPINK2-hSeq-Ex4-F	GTGGGGACTTTCACCCTCTT	423 bp
SPINK2-hSeq-Ex4-R	GCAAAAGCCAAGAAACAAGG	

Appendix Table S4. Sequence of the primer used in RT-PCR and size of the amplified products

Primer name	Primer sequence	Size of amplicon
SPINK2-hRT-F	CTGCTCCTGGCAGTTACCTT	221 bp
SPINK2-hRT-R	CAGGGTCCATTTCGAATGAT	

Appendix Table S5. Sequence of the primer used in real time RT-PCR and size of the amplified products

Primer name	Primer sequence	Size of amplicon
SPINK2-hqRT-F	CACTTTAACCCTGTGTGTGG	117 bp
SPINK2-hqRT-R	TCAGCAGGGTCCATTTCGAA	
Spink2-mqRT-F	CTCATGAGACTCTCGACTCTTCC	150 bp
Spink2-mqRT-R	TACATTCATTGCTGTAAGTGTTCATATC	
ACTB-hqRT-F	CCAACCGCGAGAAGATGA	97 bp
ACTB-hqRT-R	CCAGAGGCGTACAGGGATAG	
Actb-mqRT-F	ACCAGAGGCATACAGGGACA	104 bp
Actb-mqRT-R	CTAAGGCCAACCGTGAAAAG	

Appendix Table S6. Sequence of the primer used for genotyping and size of the amplified products.

Primer name	Primer sequence	Size of amplicon
Spink2-WT-F	GCAATGGGCGTATCTCAAAT	174 bp
Spink2-WT-R	GGGACCTGATTTTCATGCAC	
Spink2-KO-F	CTCTTCCTCGCTCCCTTCTT	213 bp
Spink2-KO-R	GGGATTCTCCCAATCTCTCC	

Appendix Figure S1. Expression of *SPINK2* mRNA in different organs in human and mouse.

The expression pattern of SPINK2 transcript in various human **(A)** and mouse **(B)** tissues was determined by Quantitative real-time RT-PCR. Values were normalized to the expression level of beta-actin via the 2-ΔΔCT method. The 2-ΔΔCT value was set at 0 in heart, resulting in an arbitrary expression of 1. Data are presented as mean ± standard error mean (n=3). Statistical differences were assessed using t-test.

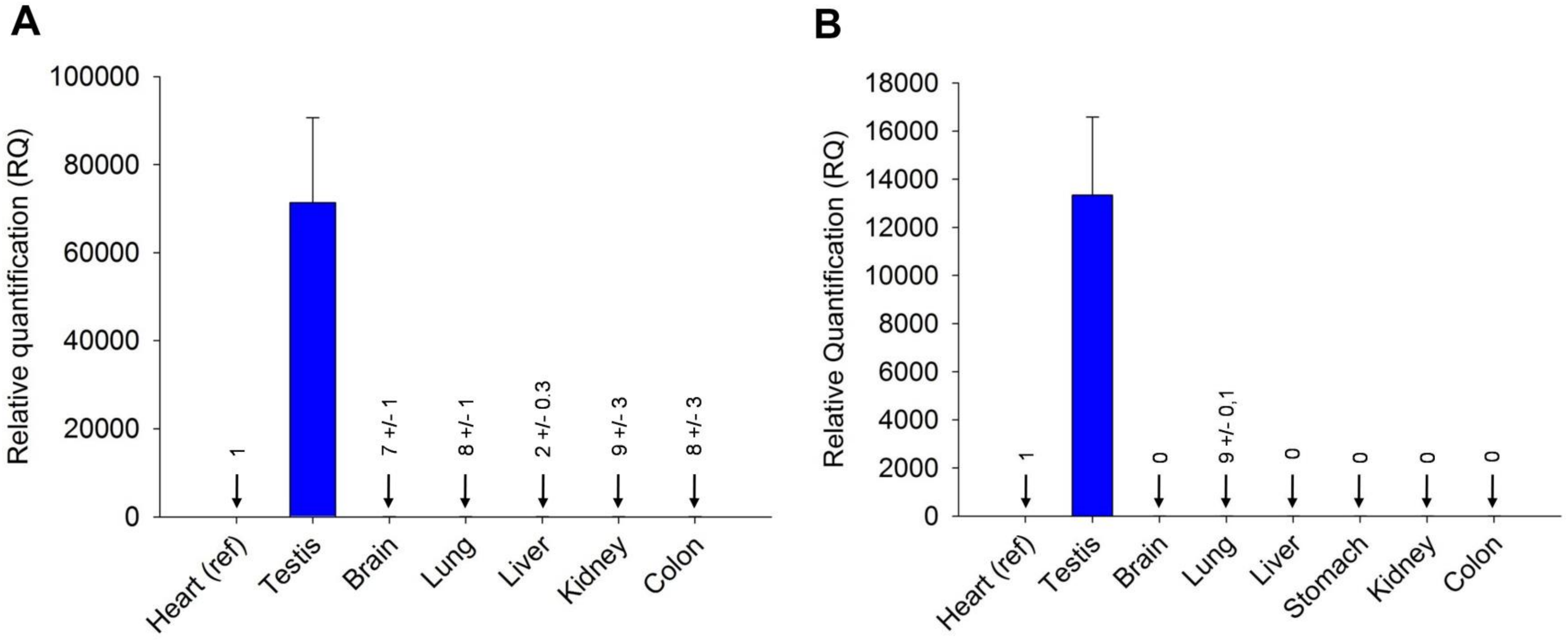

Appendix Figure S2. Impact on the protein structure of the c.206-3C>G *SPINK2* variant
(A) The normal transcript encodes for an 84 amino acids protein predicted to contain a signal peptide at the N-terminus region (blue) and a Kazal-type serine protease inhibitor domain at the C-terminus region (orange and red). UniProtKB accession number for human SPINK2 is P20155. **(B)** The c.206-3C>G splice variant generates two abnormal transcripts, T1 and T2 (see figure 2). Translation of these transcripts is predicted to produce truncated proteins lacking the Kazal-type serine protease inhibitor domain.

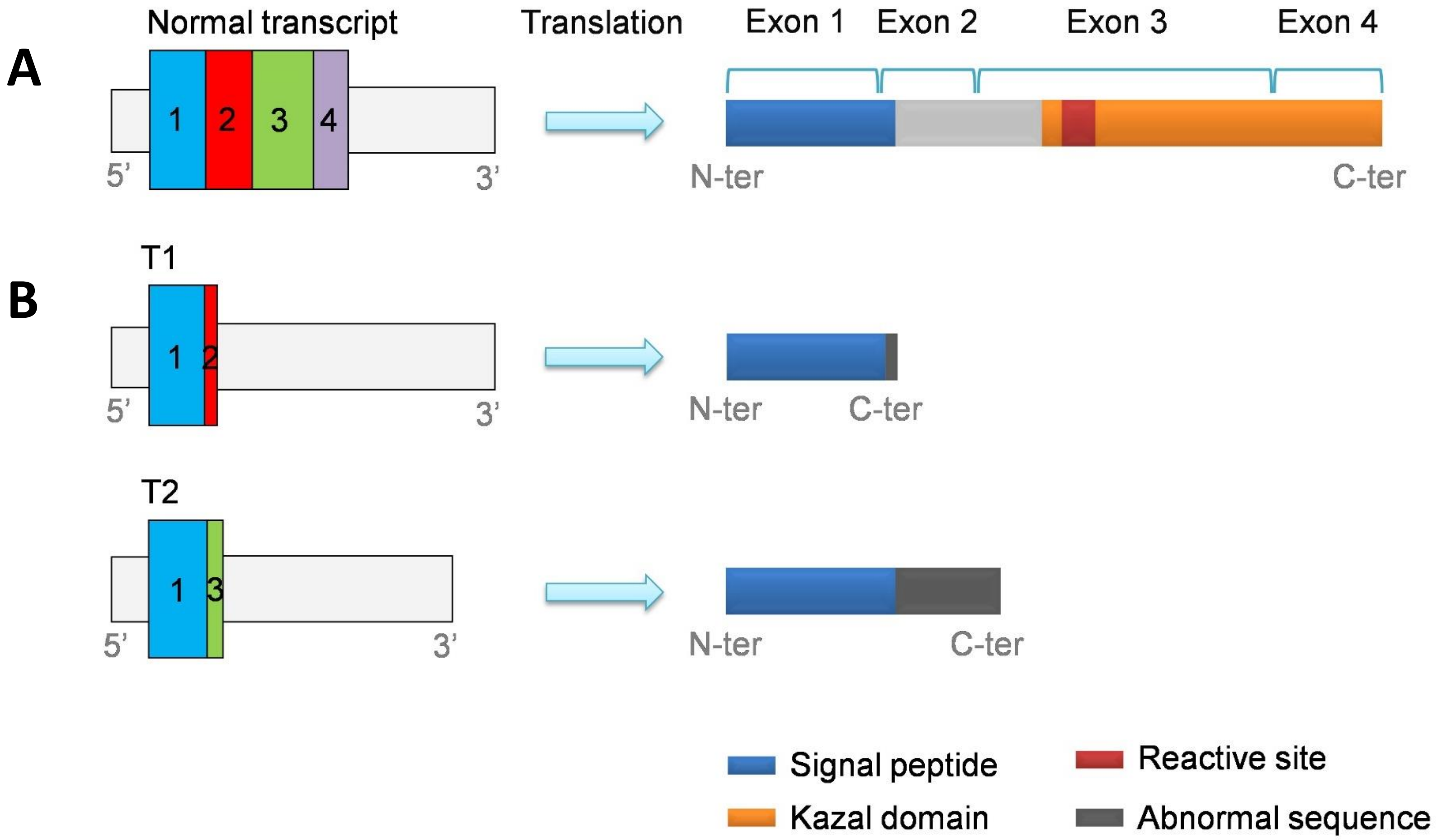

Appendix Figure S3. Absence of *Spink2* expression in testicular extracts from KO mice.
Relative quantification of *Spink2* transcript in testicular cells from **(A)** WT mice (n=3) and **(B)** *Spink2* $^{-/-}$ mice was determined by quantitative real-time RT-PCR. Values were normalized to the expression level of beta-actin via the 2-ΔΔCT method. The 2-ΔΔCT value was set at 0 in liver cells from WT mice (n=3), resulting in an arbitrary expression of 1. Data are presented as mean ± standard error mean. Statistical differences were assessed using t-test.

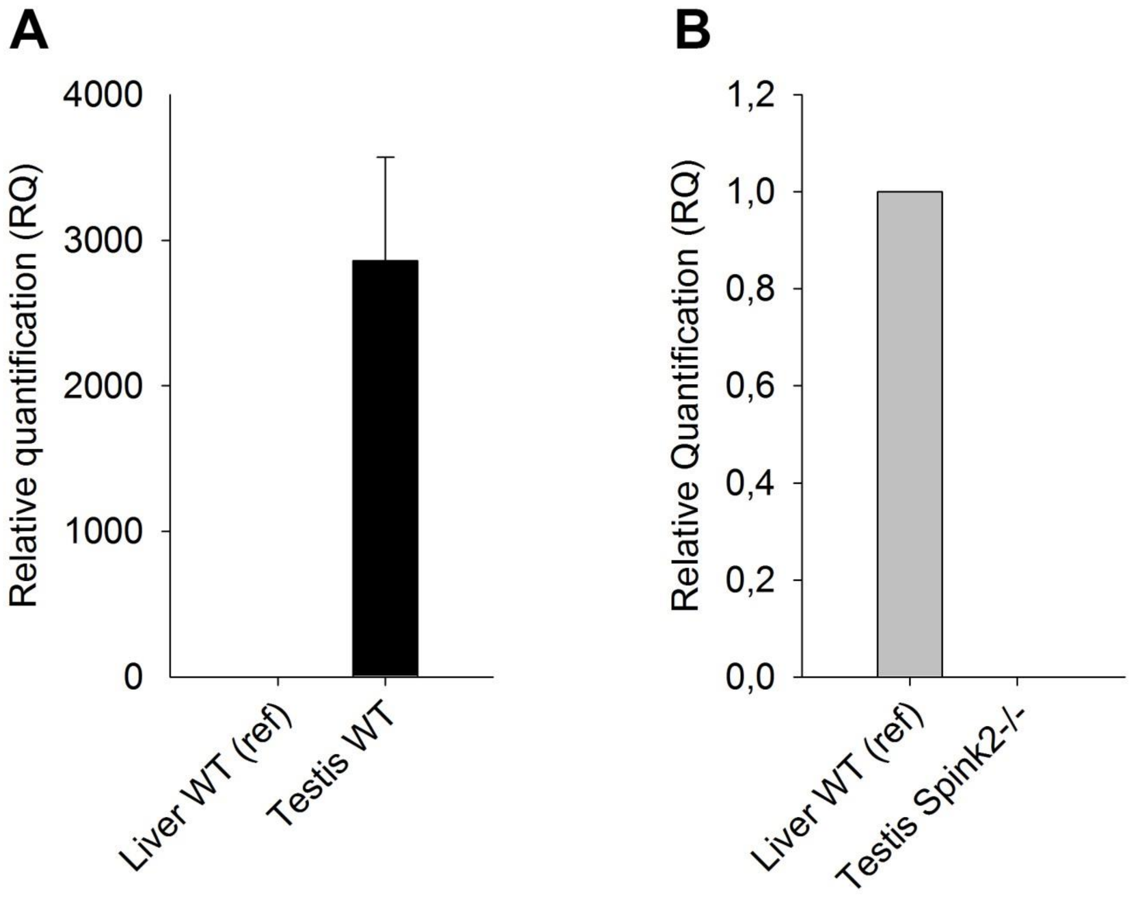

Appendix Figure S4. Specificity of antibodies targeting SPINK2.
(A) Western blot of protein extracts from HEK 293 cells heterologously transfected with or without a plasmid containing human isoform of SPINK2 tagged with DDK and revealed with an anti-SPINK2 antibody. **(B)** Similar experiment but revealed with an anti-DDK antibody. **(C)** Confocal images of HEK 293 cells transfected with human SPINK2-DDK and stained with an anti-SPINK2 antibodies (green) (top). All cells were counterstained with Hoechst (blue, staining) to mark the nuclei. Only transfected cells showed specific staining. Bottom, confoncal images of control non transfected cells showing no staining.

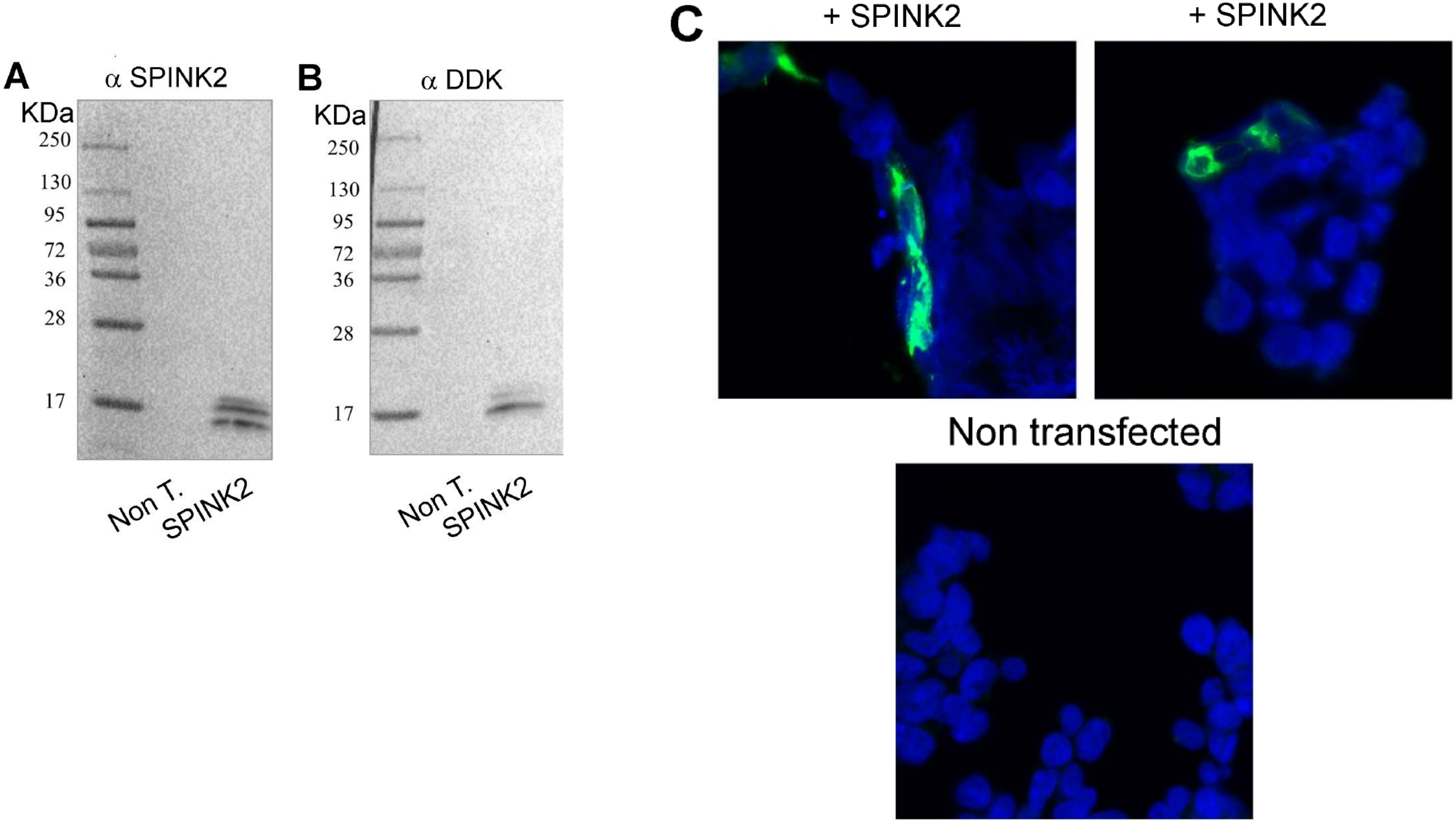

Appendix Figure S5. Absence of Spink2 does not lead to DNA fragmentation in spermatogenic cells.
(A) Representative images of TUNEL staining (green) counterstained with Hoechst (blue) of WT and *Spink2*$^{-/-}$ seminiferous tubule. Scale bar 70 µm. (**B**) Graph showing the % of cells with non fragmented DNA in WT (99.6 +/- 0.3 mean +/- SD, number of cells assessed 230-270 per biological replicate, n=3) and in *Spink2*$^{-/-}$ (99.6 +/- 0.1 mean +/- SD, number of cells assessed 300-380 per biological replicate, n=4) seminiferous tubules. NS "not statistically significant" (t-test) (**C**) Representative images of TUNEL staining (green) counterstained with Hoechst (blue) of fertile control and patient B1 seminiferous tubule. Scale bar 70 µm. (**D**) Graph showing the % of cells with non fragmented DNA in fertile control (number of cells assessed 812, n=1) and patient B1 control (number of cells assessed 789, n=1) seminiferous tubules.

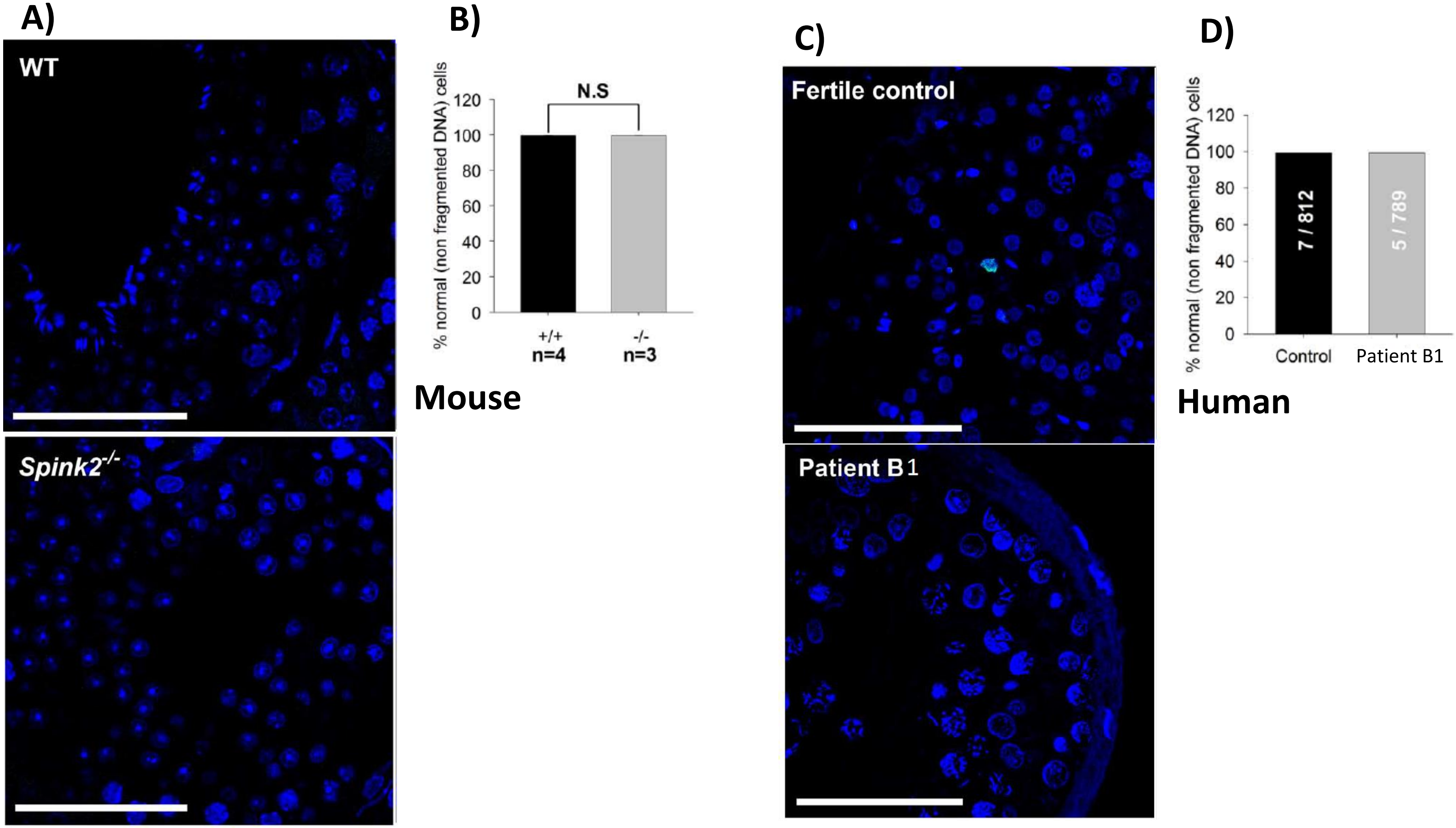

Appendix Figure S6. Equal protein loading was verified by stain-free gel technology and Western blots against house keeping protein **(A)** Stain-free^{TM} technology was used to control protein loading levels. Left, activated gel showing loading of Western-blot of Fig 9D and right of Fig 9E (**B**) Identical loading as tested in figure 9D and 9D were revealed by anti-tubulin antibody and confirmed equal protein loading between different lanes.

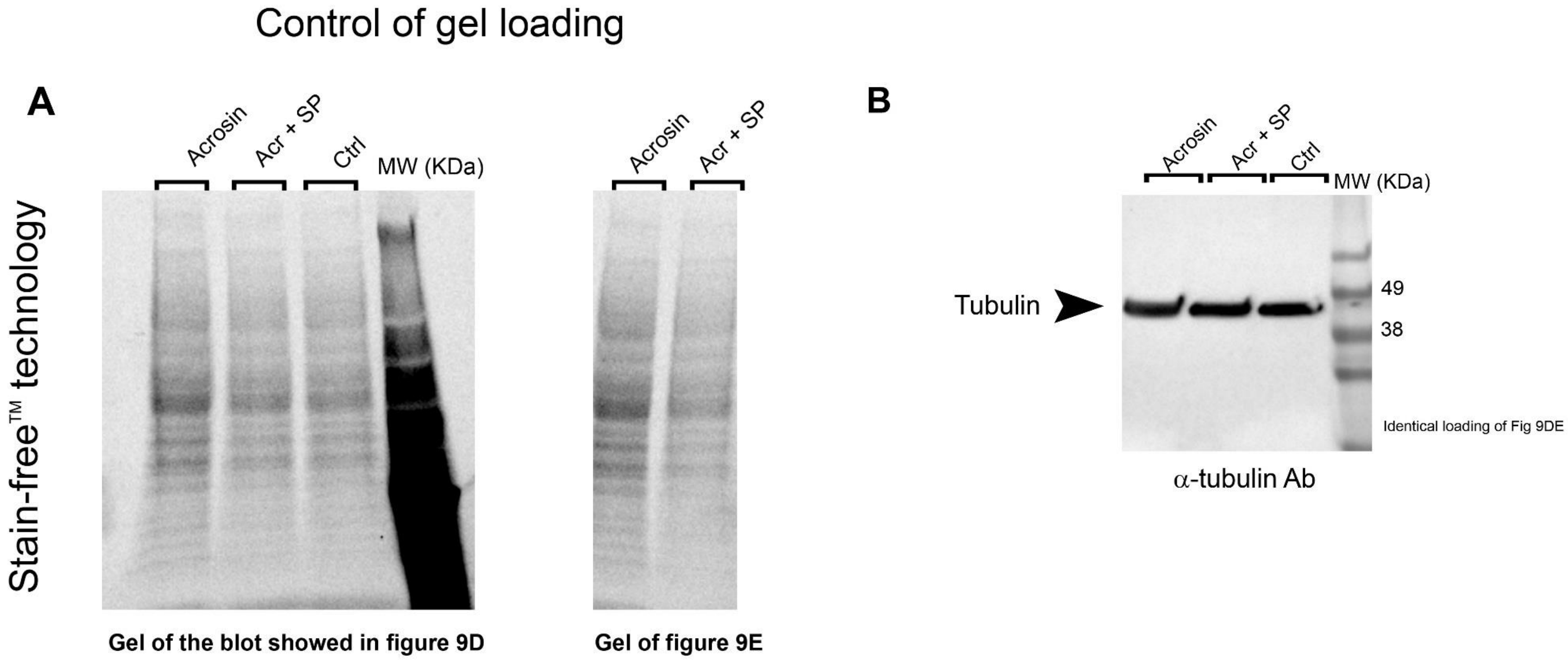

Supplement: Supplementary file 1 — Appendix [file EMMM-9-1132-s001.pdf]
