# Supplementary material for: SPINK2 deficiency causes infertility by inducing sperm defects in heterozygotes and azoospermia in homozygotes
Source: EMBO Mol Med. 2017 May 29;9(8):1132–49. doi: 10.15252/emmm.201607461 (PMC5538632; doi:10.15252/emmm.201607461)
Supplement: Supplementary file 2 — Expanded View Figures PDF [file EMMM-9-1132-s002.pdf]

Expanded View Figures

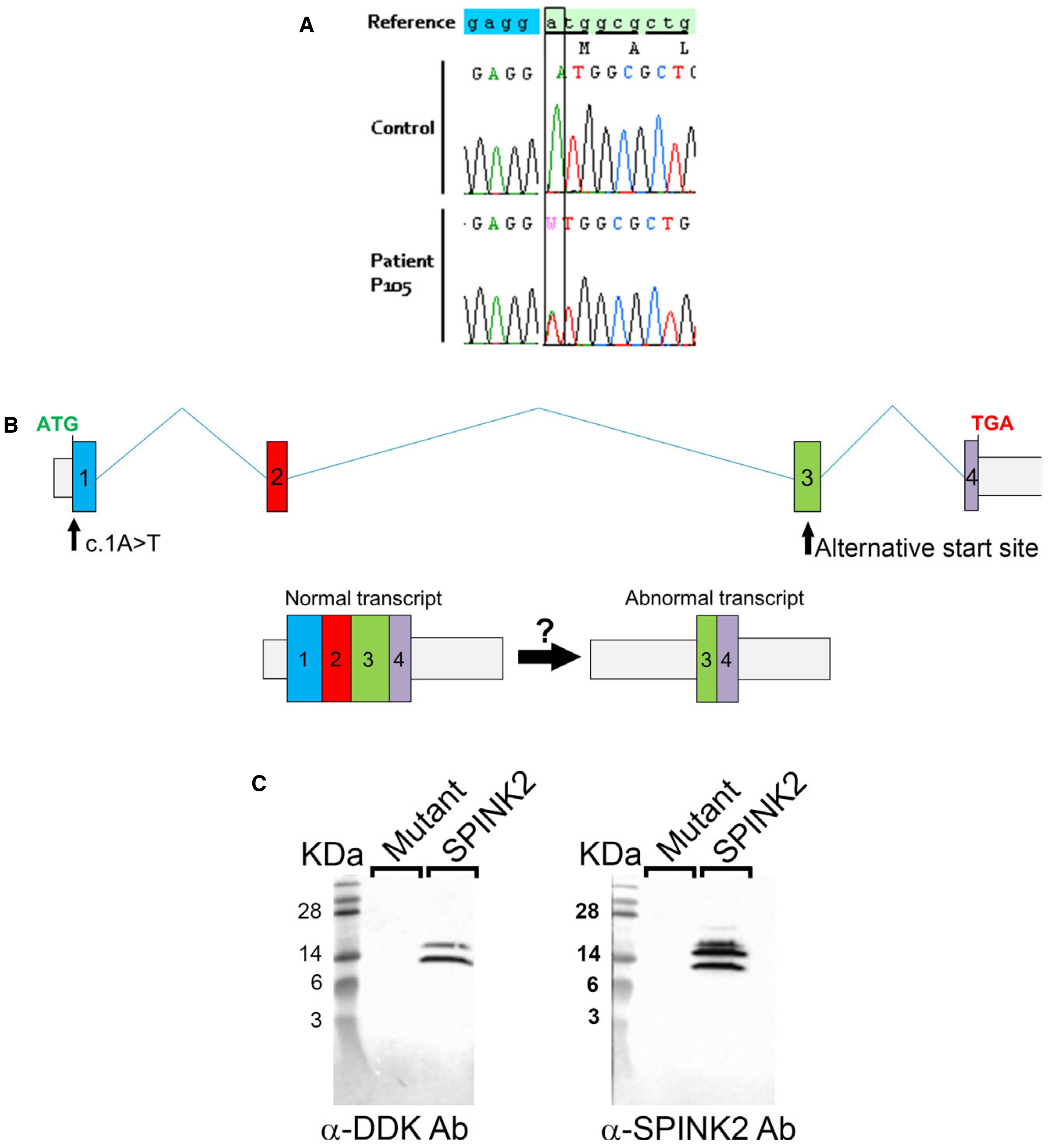

Figure EV1. Genetic analyses of the c.1A>T variant identified in patient P105.

A The c.1A>T variant, heterozygous in patient P105, abrogates the original start codon.

B An alternative start codon is present in the middle of exon 3 and may initiate translation to potentially produce a protein containing the last 27 amino acids of the wild-type protein.

C Western blot of HEK293 cell extracts transfected with C-terminus DDK-tagged SPINK2 or c.1A>T SPINK2 mutant and revealed by anti-DDK or anti-SPINK2 antibodies shows the absence of the putative truncated SPINK2 form.

© 2017 The Authors

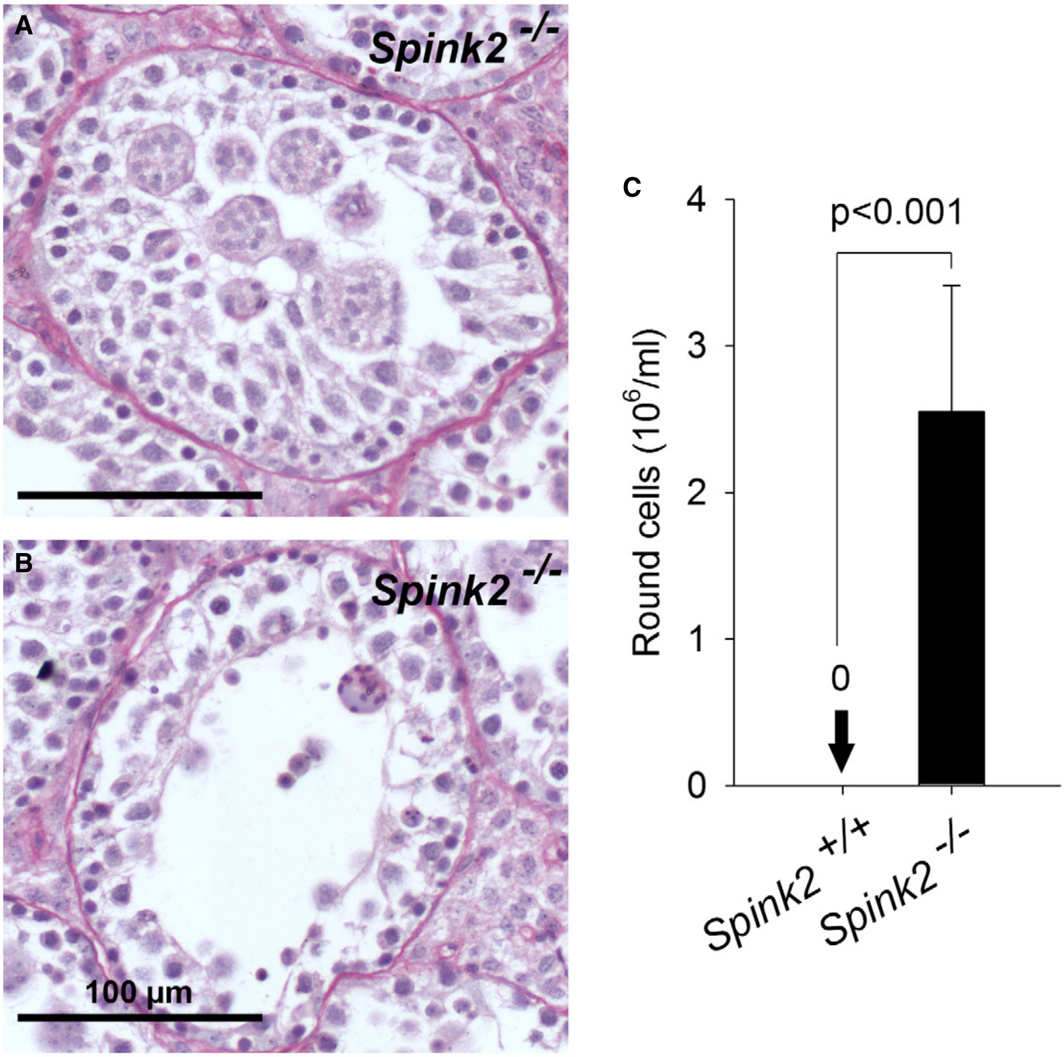

Figure EV2. Lack of Spink2 leads to germ cell desquamation and the presence of round cells in the cauda epididymis.

A, B Staining with hematoxylin and eosin of seminiferous tubule sections from *Spink2*$^{-/-}$ mice showed the presence of numerous round cells or symblasts within the lumen of tubules. Note that photograph (A) corresponds to a close-up of part of Fig 3C1 as it nicely illustrates the presence of symblasts in the tubule. Scale bar, 100 μm.

C Spermatocytograms of cauda epididymis extracts revealed the presence of only round cells at concentrations above 2 million/ml, whereas such cells were absent in WT extracts. Data are presented as mean ± SEM (n = 5).

 © 2017 The Authors

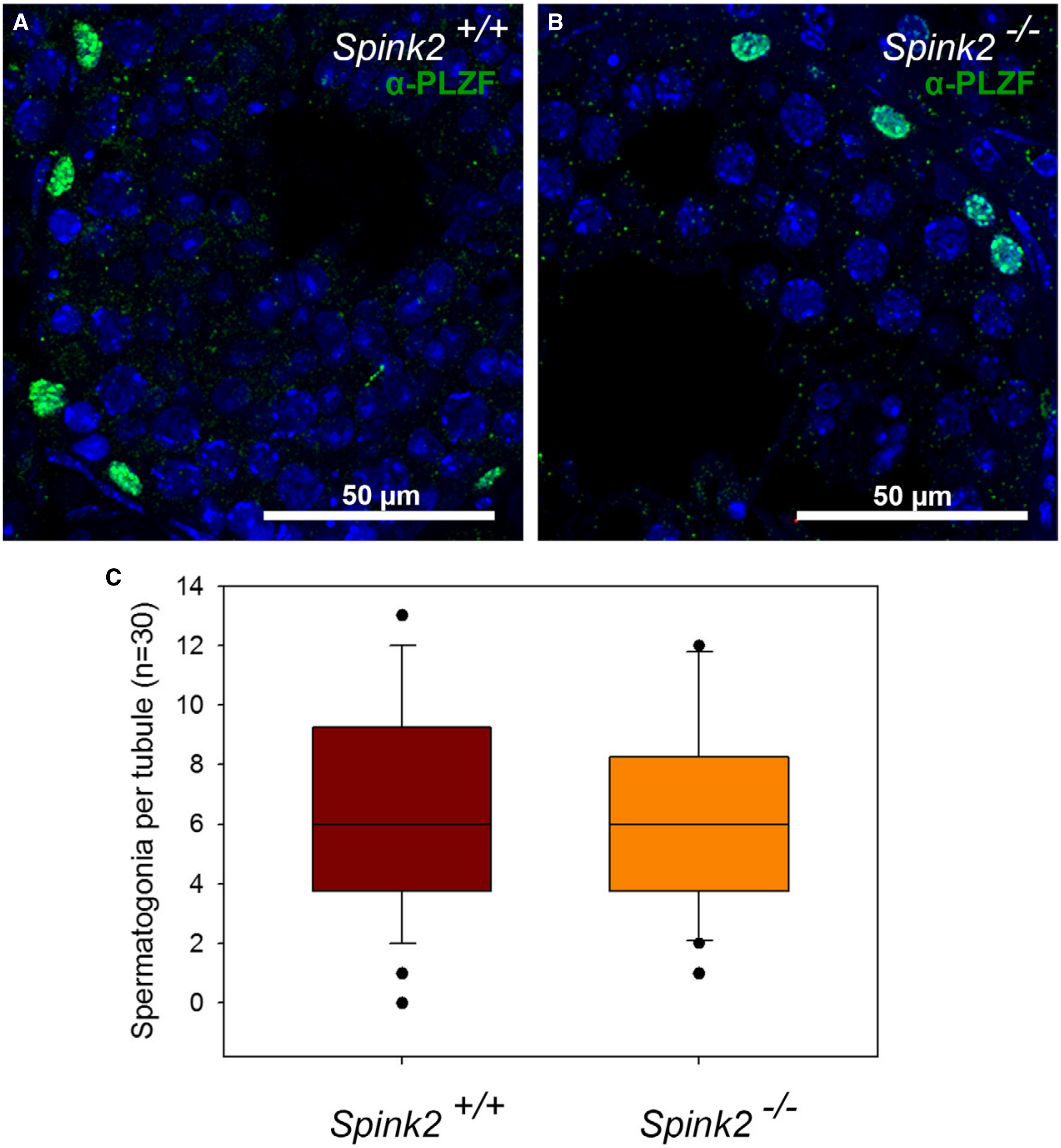

Figure EV3. Pool of non-differentiated spermatogonia is conserved in seminiferous tubules of *Spink2*$^{-/-}$ mice.

A, B Identification of spermatogonia by IF experiments using an anti-PLZF antibody in WT and *Spink2*$^{-/-}$ seminiferous tubule sections, respectively. Scale bars as indicated.

C Box plot showing the number of spermatogonia per seminiferous tubule section (n = 30 for each). No difference in the number of spermatogonia per seminiferous tubule section was observed. The line within the box marks the median, whiskers (error bars) above and below the box indicate the 90th and 10th percentiles, and black dots represent outliers.

© 2017 The Authors

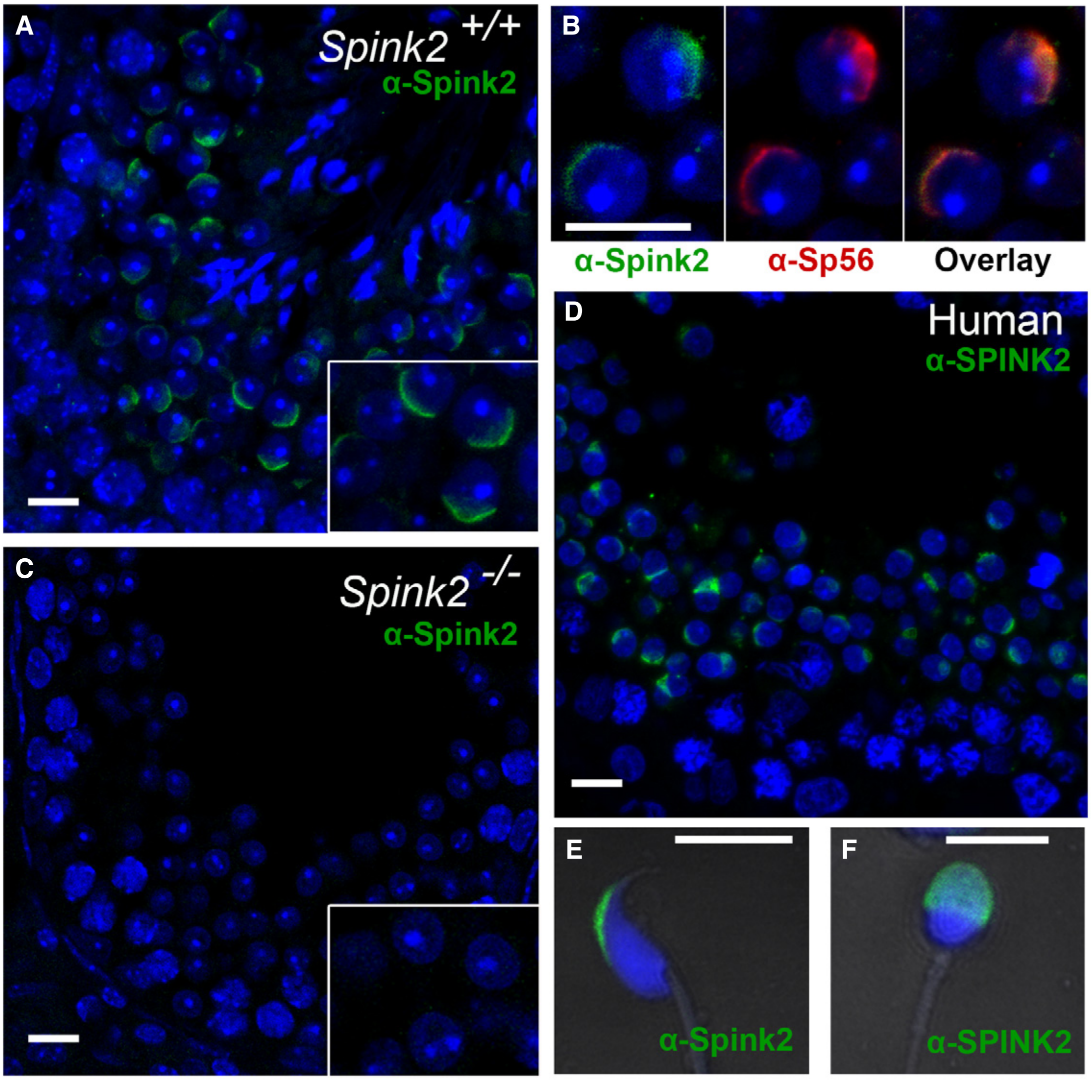

Figure EV4. SPINK2 is located in the acrosome of round spermatids and mature spermatozoa in mouse and human.

A Immunofluorescence experiments using an anti-SPINK2 antibody (green staining) reveals that Spink2 is located in the acrosome of WT mouse round spermatids. Scale bar, 10 μm.

B A double staining using an anti-SPINK2 antibody (green staining) and an anti-Sp56 antibody (red staining) shows that the two signals are colocalized. Scale bar, 10 μm.

C No Spink2 staining is observed in seminiferous tubule sections from *Spink2*$^{-/-}$ mice, stained with an anti-SPINK2 antibody. Scale bar, 10 μm.

D In sections of human seminiferous tubules, a similar localization of SPINK2 is observed in round spermatids, corresponding to the acrosome. Scale bar, 10 μm.

E IF experiments using an anti-SPINK2 antibody (green staining) reveal that Spink2 is located in the acrosome of WT epididymal mouse sperm. Scale bar, 10 μm.

F SPINK2 localization within the acrosome is conserved in ejaculated human sperm. No staining is observed in the midpiece or principle piece. Scale bar, 5 μm.

© 2017 The Authors

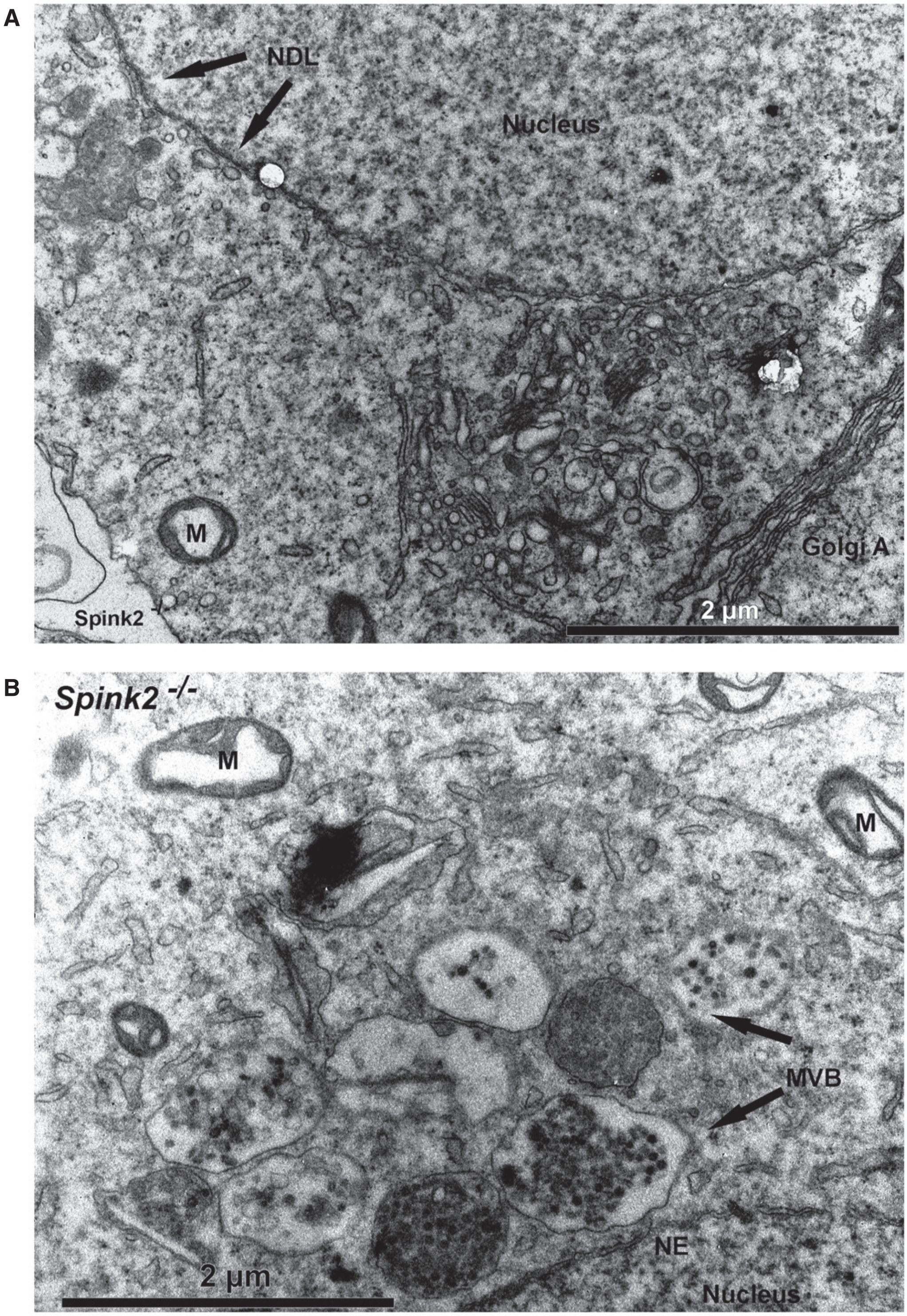

Figure EV5. Disjunction of the polarity of the Golgi apparatus and the nuclear dense lamina (NDL) in *Spink2*$^{-/-}$ round spermatids and presence of multivesicular bodies in the cytoplasm of *Spink2*$^{-/-}$ round spermatids.

A Partial section of a *Spink2*$^{-/-}$ round spermatid observed by EM showing the spatial disjunction of the Golgi apparatus and the NDL. NDL is evidenced by apposition of dense material on the nuclear side of the inner nuclear membrane. Another hallmark of this specialized area of the nuclear envelope is the tight apposition of both inner and outer nuclear membranes along the NDL. M, mitochondria. Scale bar, 2 µm.

B Partial section of a *Spink2*$^{-/-}$ round spermatid observed by EM showing structures corresponding to multivesicular bodies (MVB). M, mitochondria; NE, nuclear envelope; NDL, nuclear dense lamina. Scale bar, 2 µm.

© 2017 The Authors
