# Supplementary material for: SPINK2 deficiency causes infertility by inducing sperm defects in heterozygotes and azoospermia in homozygotes
Source: EMBO Mol Med. 2017 May 29;9(8):1132–49. doi: 10.15252/emmm.201607461 (PMC5538632; doi:10.15252/emmm.201607461)
Supplement: Supplementary file 3 — Table EV1 [file EMMM-9-1132-s003.docx]

Table EV1 : Patient P105 sperm parameters.

|  | | **Sample 1** | **Sample 2** | **mean** | **Lower limit (WHO)^1^** |
| --- | --- | --- | --- | --- | --- |
| **Ejaculate volume, ml** | | 1.5 | 1.5 | 1.5 | 1.5 |
| **Sperm concentration (Million/ml)** | | 0.3* | 3* | 1.65* | 15 |
| **Normal forms (%)** | | 34 | 39 | 36.5 | 4 |
| **Sperm defects** | Acrosome defect (% of AS) | 32 | 28 | 30 |  |
|  | Neck-base defect (% of AS) | 40 | 46 | 43 |  |
|  | Flagellum defect (% of AS) | 26 | 26 | 26 |  |
| **Sperm defects** | Motility a 1 h (%)  rapid progressive motility | 5* | 0* | 2,5* | a>25% |
|  | Motility b, 1 h (%)  slow progressive motility | 45 | 35 | 40 | a+b 32% |
|  | Motility d, 1 h (%)  immotility | 45 | 60 | 52.5 | a+b+c 40% |

**Table EV1 - Spermatocytograms of patient P105 presenting a heterozygous mutation in *SPINK2* and presenting with astheno-oligozoospermia.**

“% of AS” means percentage of abnormal sperm. * corresponds to values below the lower acceptable values.^1.^ World Health Organization (2010). *WHO laboratory manual for the Examination and processing of human semen -Fifth edition*.
